# *Fusarium* Mycotoxins Zearalenone and Deoxynivalenol Reduce Hepatocyte Innate Immune Response after the *Listeria monocytogenes* Infection by Inhibiting the TLR2/NFκB Signaling Pathway

**DOI:** 10.3390/ijms24119664

**Published:** 2023-06-02

**Authors:** Nannan Feng, Fang Zhong, Guodong Cai, Wanglong Zheng, Hui Zou, Jianhong Gu, Yan Yuan, Guoqiang Zhu, Zongping Liu, Jianchun Bian

**Affiliations:** 1 College of Veterinary Medicine, Yangzhou University, Yangzhou 225009, China; dx120190150@yzu.edu.cn (N.F.); fzhong2022@outlook.com (F.Z.); 008400@yzu.edu.cn (G.C.); wanglongzheng@yzu.edu.cn (W.Z.); zouhui@yzu.edu.cn (H.Z.); jhgu@yzu.edu.cn (J.G.); yuanyan@yzu.edu.cn (Y.Y.); yzgqzhu@yzu.edu.cn (G.Z.); liuzongping@yzu.edu.cn (Z.L.); 2 Joint International Research Laboratory of Agriculture and Agri-Product Safety of the Ministry of Education of China, Yangzhou University, Yangzhou 225009, China; 3 Jiangsu Co-innovation Center for Prevention and Control of Important Animal Infectious Diseases and Zoonoses, Yangzhou 225009, China

**Keywords:** zearalenone, deoxynivalenol, *Listeria monocytogenes*, immunotoxicity, TLR2/NFκB signaling

## Abstract

Zearalenone (ZEA) and deoxynivalenol (DON) are two common mycotoxins produced by the genus *Fusarium* and have potential immunotoxic effects that may lead to a weak immune response against bacterial infections. *Listeria monocytogenes* (*L. monocytogenes*), a food-borne pathogenic microorganism ubiquitous in the environment, actively multiplies in the liver, where hepatocytes are capable of resistance through mediated innate immune responses. At present, it is not clear if ZEA and DON affect hepatocyte immune responses to *L. monocytogenes* infection or the mechanisms involved. Therefore, in this study, in vivo and in vitro models were used to investigate the effects of ZEA and DON on the innate immune responses of hepatocytes and related molecules after *L. monocytogenes* infection. In vivo studies revealed that ZEA and DON inhibited the toll-like receptors 2 (TLR2)/nuclear factor kappa-B (NFκB) pathway in the liver tissue of *L. monocytogenes*-infected mice, downregulating the expression levels of Nitric oxide (NO), in the liver and repressing the immune response. In addition, ZEA and DON inhibited Lipoteichoic acid (LTA)-induced expression of TLR2 and myeloid differentiation factor 88 (MyD88) in Buffalo Rat Liver (BRL 3A) cells in vitro, downregulating the TLR2/NFκB signaling pathway and resulting in the decreased expression levels of NO, causing immunosuppressive effects. In summary, ZEA and DON can negatively regulate NO levels through TLR2/NFκB, inhibiting the innate immune responses of the liver, and aggravate *L. monocytogenes* infections in mouse livers.

## 1. Introduction

ZEA and DON, as two common mycotoxins produced by *Fusarium*, contaminate food and feed all over the world and seriously threaten human and animal health. ZEA, also known as F-2 toxin, is a non-steroidal estrogenic mycotoxin with a wide range of toxic effects, including cytotoxicity, reproductive toxicity, genotoxicity, and immunotoxicity [1]. DON is a trichothecene type B toxoid, also known as vomitoxin, with enterotoxicity, neurotoxicity and immunotoxicity [2,3]. Although the main toxicities were induced by ZEA in reproductive tissues [4] and by DON in enterotoxicity [5], their potential immunosuppressive effects cannot be ignored. Cai et al. [6,7] reported that ZEA can inhibit the activation of T lymphocytes and B lymphocytes cultured in vitro and the chemotaxis of T cells, while DON can also inhibit the immune function of T cells and induce cell apoptosis. Subsequently, Cai et al. [8] found that ZEA and DON decreased pro-inflammatory cytokines interleukin-1β (IL-1β) and interleukin-12 (IL-12) and inhibit NF-kB signaling after infection with *L. monocytogenes* in the spleen. However, the effects of ZEA and DON on immune responses in non-central and non-peripheral immune organs such as the liver are less studied and deserve further investigation.

The immunosuppressive effect caused by mycotoxin may be reflected in the process of pathogen infection. *L. monocytogenes* is a gram-positive facultative anaerobic bacterium that is widespread in the environment [9,10]. There are currently 17 known species of Listeria, and *L. monocytogenes* is considered the most pathogenic among them. It is known to cause several human and animal diseases [11,12]. Human infections are caused by contaminated food, including raw vegetables, raw milk, fish, and poultry, and pregnant women are especially vulnerable, as *L. monocytogenes* can infect the fetus [13]. Animal infections are usually caused by contaminated feed, especially silage or other foods contaminated with *L. monocytogenes*, and most wild and domestic animals are susceptible to *L. monocytogenes* when ingested [14]. The liver is the main site of *L. monocytogenes* infection and proliferation, Gaillard et al. [15] studied the virulence of an inlAB mutant and of its parent in murine *L. monocytogenes*. Their findings indicated that the inlAB protects *L. monocytogenes* from the host’s innate defense mechanisms by promoting its entry into hepatocytes. Shen et al. [16] found that the virulent *L. monocytogenes* replicates primarily in the liver after infecting hepatocytes, however, bacterial entry requires an interaction of InlAB with Mesenchymal-epithelial transition (MET) affected pathogenesis. Nitric oxide (NO) produced by hepatocytes is important for innate immune responses, as it can inhibit nucleotide reductase in bacteria, reducing bacterial DNA synthesis and inducing bacterial DNA double-strand breaks to prevent bacterial replication [17]. Furthermore, NO can increase bacterial susceptibility to DNA oxidative damage by blocking bacterial respiration [18].

After *L. monocytogenes* infects the liver, inducing cell innate immunity provides an immediate response, which is nonspecific and generalized to injury or infection. The response is mediated by germline-encoded pattern recognition receptors, such as Toll-like receptors (TLRs) [19]. TLR2 is a key innate immune receptor. David et al. [20] observed that TLR2-deficient mice were more susceptible to systemic infection with *L. monocytogenes* than wild-type mice, causing reduced survival rates and an increased bacterial load in the liver [19,20]. When the body is infected with pathogenic microorganisms, such as Gram-positive pathogens, hepatocytes can produce NO through the TLR2/NFκB pathway as a part of innate immunity [21]. Zhao et al. showed that a selenium (Se) deficiency led to inflammation by activating the NF-κB pathway through multiple mechanisms in the livers of pigs, causing immune responses [22]. The TLR2/NFκB signaling pathway’s role in the innate immune response to bacterial infection in conjunction with mycotoxins in the liver is currently unclear.

To further understand the role of mycotoxins in the innate immune response of the liver, we investigated if bacterial infection, in conjunction with ZEA and DON, had an effect on the innate immune response of the liver both in vitro and in vivo, as well as its effects on the TLR2/NFκB signaling pathway. This provides a basis for further research on the effects of ZEA and DON immunotoxicity regarding the immune response after foodborne bacterial infection.

## 2. Results

### 2.1. ZEA and DON Can Inhibit the Viability of BRL 3A Cells

**Figure 1:**
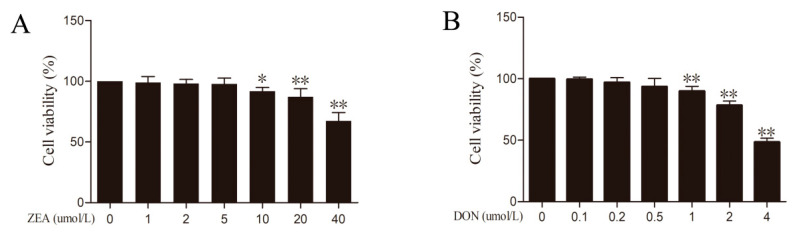
Effects of ZEA and DON on BRL 3A cells viability. CCK-8 was used to detect the effect of ZEA (**A**) and DON (**B**) on BRL 3A cells viability. Values are the mean ± SD of three individual experiments; * *p* < 0.05, ** *p* < 0.01 indicate significant differences between toxins treated groups and control groups.

Cell viability was detected via a cell counting kit-8 (CCK-8) assay. The data from the CCK-8 (Figure 1) showed that the viability of the BRL 3A cells was decreased in a dose-dependent manner after treatment with different concentrations of ZEA and DON for 24 h. ZEA caused a significant decrease in cell viability at 10 μmol/L (*p* < 0.05) and showed a dose-dependent effect (Figure 1A). DON caused a significant decrease in cell viability at 1 μmol/L (*p* < 0.05) and showed a dose-dependent effect (Figure 1B). In the following test, 10 μmol/L and 20 μmol/L of ZEA and 1 μmol/L and 2 μmol/L of DON, were selected as the concentration for the further study, respectively.

### 2.2. ZEA and DON Inhibited Gene and Protein Expression of TLR2 and MyD88 in All BRL 3A Cells 

**Figure 2:**
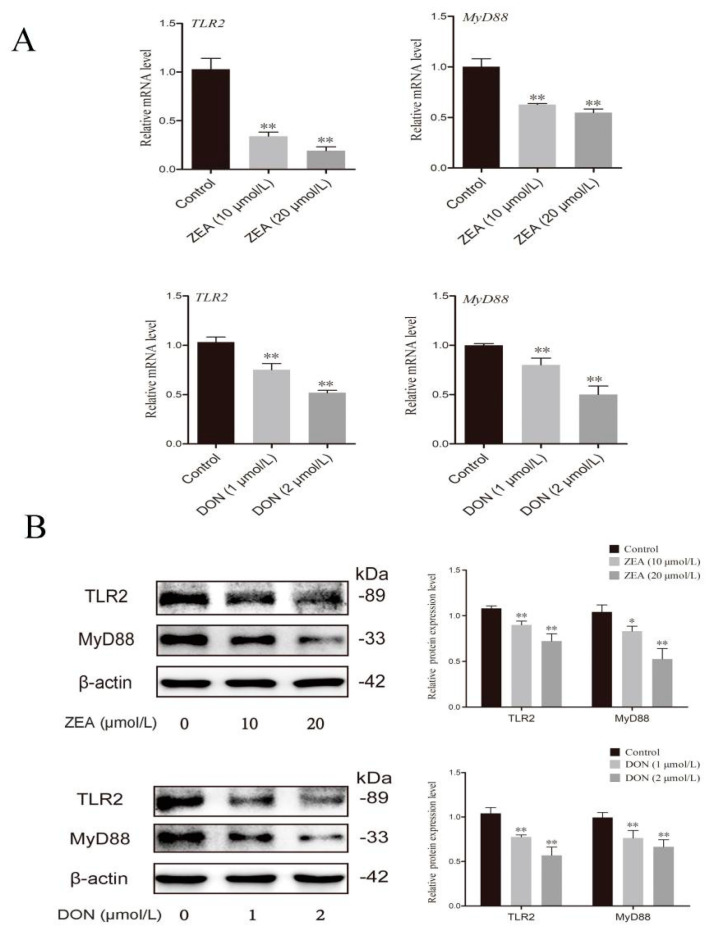
Effect of ZEA and DON exposures on the mRNA and protein expressions of the TLR2 and MyD88 in BRL 3A cells. (**A**) qPCR detected the mRNA levels of TLR2 and MyD88. (**B**) The expressions of TLR2 and MyD88 were estimated via western blot. Values are the mean ± SD of three individual experiments. * *p* < 0.05, ** *p* < 0.01 indicate significant differences between toxin treated groups and control groups.

TLR2 is a receptor involved in the cellular recognition of Gram-positive bacteria. It transmits a signal downstream through the receptor adaptor protein myeloid differentiation factor (MyD88), causing the release of immune products. The effect of ZEA and DON on the expression of TLR2 and MyD88 in BRL 3A cells was measured (Figure 2), revealing that ZEA and DON both significantly inhibited both gene (Figure 2A) and protein (Figure 2B) expression of TLR2 and MyD88 (*p* < 0.05 or *p* < 0.01).

### 2.3. ZEA and DON Inhibit LTA-Induced BRL 3A Cells NOS2 Expression and NO Production

**Figure 3:**
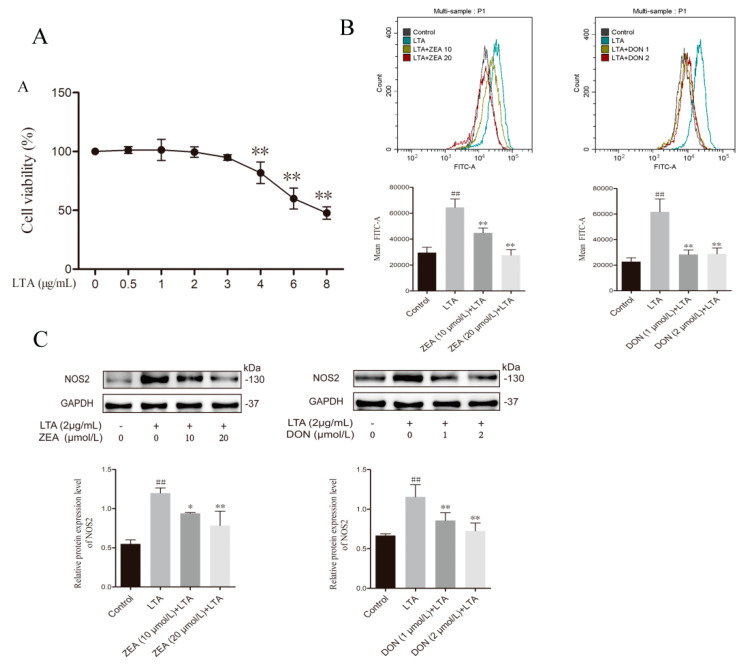
Effects of ZEA and DON on NO production and NOS2 expression after LTA treatment of BRL 3A cells. (**A**) Effects of LTA (0, 0.5, 1, 2, 3, 4, 6, 8 μg/mL) treated on BRL 3A cell viability, compared to the control group, ** *p* < 0.01. (**B**) Flow cytometry detected intercellular levels of NO. (**C**) The proteins expression levels of NOS2 by Western Blot. Values are the mean ± SD of three individual experiments. ## *p*  <  0.01, indicate significant differences between LTA groups and control groups; * *p* < 0.05 and ** *p* < 0.01, indicate significant differences between toxin treated groups and LTA groups.

To further investigate the effects of ZEA and DON on BRL 3A cell immune response, Lipoteichoic acid (LTA) was used to induce immune responses in BRL 3A cells in vitro (Figure 3A). The optimum concentration of LTA was determined to be 2 μg/mL using CCK8 cells. NO is an antibacterial effector of the innate immune system and synthesized in hepatocytes via nitric oxide synthase 2 (NOS2) in the liver. NO levels and NOS2 expression were measured in response to treatment with ZEA and DON, as well as LTA treatment in BRL 3A cells via flow cytometry and immunoblotting. After LTA stimulation, the cellular NO levels and the NOS2 protein expression level increased significantly (*p* < 0.01). Compared with the LTA group, both the ZEA and DON groups had significantly decreased NO concentrations and NOS2 protein expression (*p* < 0.05 or *p* < 0.01) (Figure 3B,C), suggesting that ZEA and DON could both inhibit the production of NO.

### 2.4. ZEA and DON Inhibited the Activation of the TRL2/NFκB Signaling Pathway in BRL 3A Cells Induced by LTA

**Figure 4:**
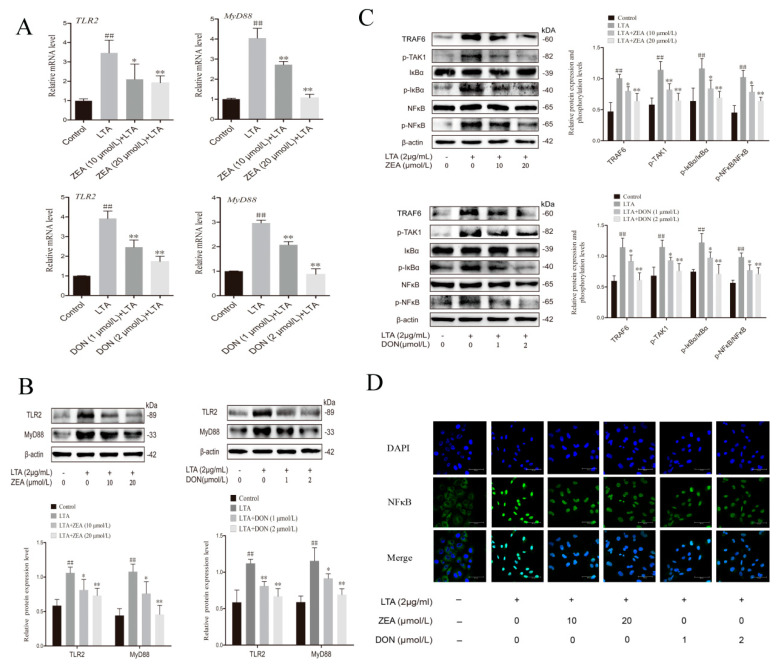
ZEA and DON inhibited the LTA-induced TLR2/NFκB signaling pathway in BRL 3A cells. (**A**,**B**) The gene and protein expression of TLR2 and MyD88 was detected via qPCR and Western blotting. (**C**) Protein expression and phosphitylation levels of TRAF6, p-TAK1, p-IκBα/IκBα, and p-NFκB/NFκB were analyzed via western blotting. Values are the mean ± SD of three individual experiments. ## *p*  <  0.01, indicate significant differences between LTA groups and control groups; * *p* < 0.05 and ** *p* < 0.01, indicate significant differences between toxin treated groups and LTA groups (**D**) Fluorescence microscopy images indicate that NFκB nuclear translocation occurred. NFκB (green), DAPI (blue), scale bar = 50 µm.

To further explore the mechanisms by which ZEA and DON reduced LTA-induced NO levels in BRL 3A cells, expression of the TLR2/NFκB signaling pathway was measured. The gene and protein expression of TLR2 and MyD88 in the LTA group increased significantly (*p* < 0.01 or *p* < 0.05), while expression in the ZEA and DON treatment groups significantly decreased (*p* < 0.01 or *p* < 0.05) (Figure 4A,B). Subsequent measurement of pathway-related proteins revealed that ZEA and DON treatment also inhibited the expression of TNF receptor associated factor 6 (TRAF6) and phosphorylation TGF beta-Activated Kinase 1 (*p*-TAK1), as well as decreasing the phosphorylation levels of nuclear factor kappa-B (IκBα) and NFκB after LTA induction (*p* < 0.01 or *p* < 0.05) (Figure 4C). Nuclear translocation of NFκB proteins was further detected by immunofluorescence, revealing that NFκB fluorescence in the nuclei of the LTA treatment group was significantly enhanced, suggesting that more NFκB had translocated into the nucleus. NFκB fluorescence in the nuclei of cells treated with ZEA and DON decreased, indicating that the amount of protein translocated into the nucleus was reduced. This was consistent with protein measurements (Figure 4D). Overall, ZEA and DON inhibited the TLR2/NFκB signaling pathway, resulting in decreased expressions of NOS2 and decreased levels of NO.

### 2.5. Effects of ZEA and DON on Liver Histological Parameters and Bacterial Loads in L. monocytogenes-Infected Mice

**Figure 5:**
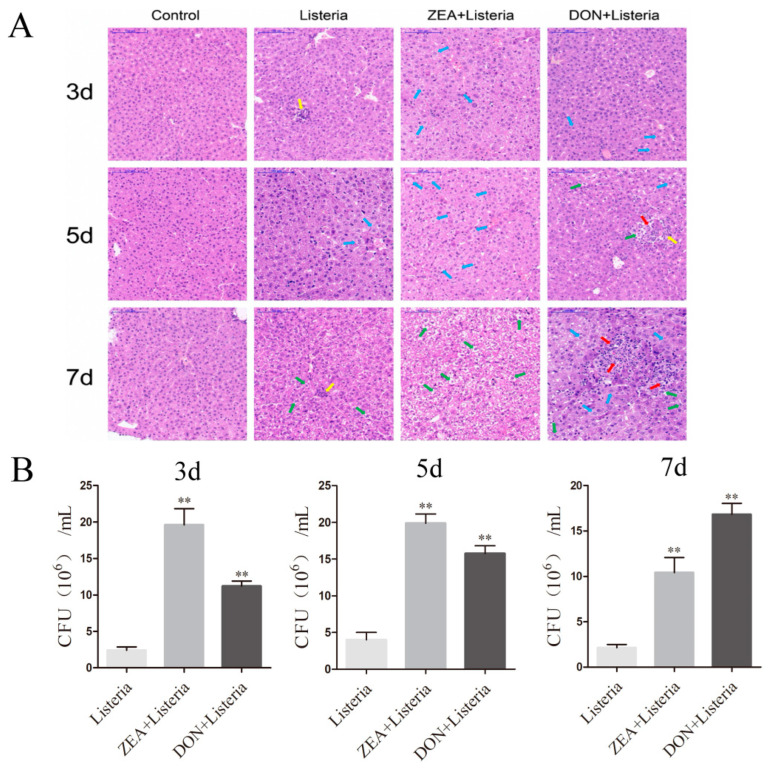
ZEA and DON aggravated liver bacterial loads and injuries in mice infected with *L. monocytogenes*. (**A**) At the third, fifth, seventh days after *L. monocytogenes* infection, liver tissue histomorphological changes (red arrows indicate nuclear fragmentation; yellow arrows indicate inflammatory cells; blue arrows indicate edema of cells with loose and lightly stained cytoplasm; green arrows indicate balloon degeneration of cells with vacuolated cytoplasm. Scale bar: 100 μm). (**B**) The bacterial burden was assessed by determining colony-forming units (CFU) numbers in the liver. Values are the mean ± SD of three individual experiments. ** *p* < 0.01 indicate significant differences between toxin treated groups and the *L. monocytogenes* group.

The pathological changes of liver tissue sections were observed via histopathological staining. On day three after bacterial infection, inflammatory cell infiltration was observed in the visceral tissue of the *L. monocytogenes* group. Compared to hepatocytes in the *L. monocytogenes* treatment group, those in the ZEA and DON treatment groups exhibited more serious edema. On day five after of bacterial infection, hepatocytes in the *L. monocytogenes* group showed edema. Compared to the hepatocytes in the *L. monocytogenes* treatment group, those in the ZEA treatment groups demonstrated severe edema, while hepatocytes in the DON treatment group demonstrated balloon degeneration. On day seven after bacterial infection, the hepatocytes in the *L. monocytogenes* treatment group began to develop balloon degeneration, whereas there were more hepatocytes and more severe balloon degeneration in the ZEA treatment group, with many cells showing cytoplasmic degeneration and observable vacuolization. In the DON treatment group, the hepatocytes were massively edematous and vacuolized with large-area necrosis (Figure 5A).

The bacterial load in the liver tissues of treated and untreated mice was detected (Figure 5B). On days three, five, and seven after bacterial infection, in comparison to the *L. monocytogenes* treatment group, the bacterial load in the liver tissues of the ZEA and DON treatment groups all increased significantly (*p* < 0.01). These results indicated that ZEA and DON increased the amount of *L. monocytogenes* in mouse livers and that the degree of bacterial infection aggravated liver damage.

### 2.6. ZEA and DON Inhibited the Expression Level of NOS2 and NO Production in the Liver of L. monocytogenes-Infected Mice

**Figure 6:**
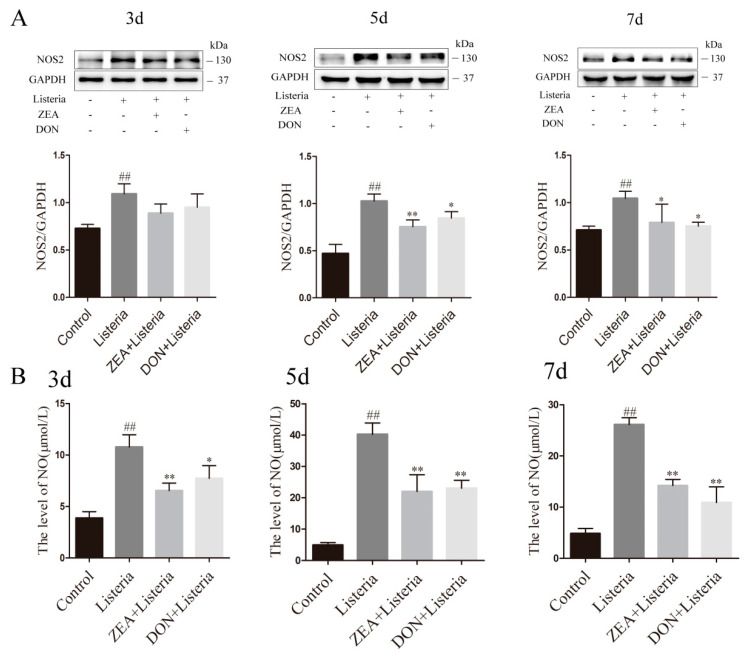
Effects of ZEA and DON on NOS2 and NO after *L. monocytogenes* infection. (**A**) The expressions of NOS2 were evaluated via western blotting. (**B**) NO changes were detectable via flow cytometry. Values are the mean ± SD of three individual experiments. ## *p*  <  0.01, indicate significant differences between the *L. monocytogenes* group and control groups; * *p* < 0.05 and ** *p* < 0.01, indicate significant differences between toxin treated groups and the *L. monocytogenes* group.

NO is synthesized in hepatocytes via nitric oxide synthase 2 (NOS2) and has an antibacterial effector. The expression levels of proteins, of NOS2, and the concentration of NO in the liver were detected at three, five, and seven days after infection with *L. monocytogenes* (Figure 6A,B). The levels of NO and NOS2 expression increased significantly (*p* < 0.05 or *p* < 0.01) after *L. monocytogenes* infection compared with the control group, indicating that the body can produce a normal antibacterial response. Compared to the *L. monocytogenes* group, NO and NOS2 levels in the ZEA and DON groups varied in degrees decreased (*p* < 0.05 or *p* < 0.01), which is consistent with the in vitro test results.

### 2.7. ZEA and DON Inhibited the TLR2/NFκB Signaling Pathway in the Liver of L. monocytogenes-Infected Mice

**Figure 7:**
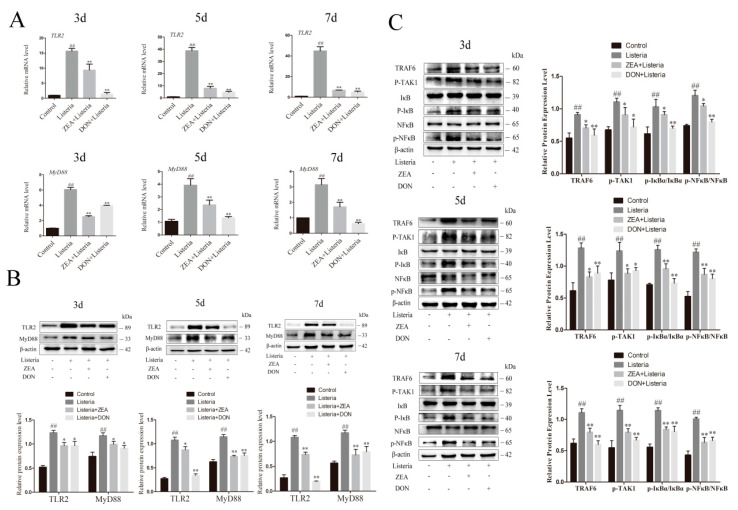
ZEA and DON can inhibit TLR2/NFκB the signaling pathway in mice infected with *L. monocytogenes*. (**A**,**B**) The mRNA and protein expression of TLR2 and MyD88 were measured by qPCR and western blotting. (**C**) Protein expression and phosphitylation levels of TRAF6, p-TAK1, p-IκBα/IκBα, and p-NFκB/NFκB. Values are the mean ± SD of three individual experiments. ## *p* <  0.01, indicate significant differences between the *L. monocytogenes* group and control groups; * *p* < 0.05 and ** *p* < 0.01, indicate significant differences between toxin treated groups and the *L. monocytogenes* group.

The innate immune responses of hepatocytes against *L. monocytogenes* infection can be exerted through the TLR2/NFκB pathway. The expression and phosphorylation levels of proteins associated with the TLR2/NFκB signaling pathway in the liver were detected at three, five, and seven days after infection with *L. monocytogenes* (Figure 7). Compared to the control group, the expression of TLR2, MyD88 mRNA, and protein was significantly increased in the *L. monocytogenes* group (*p* < 0.01). However, the protein and mRNA expression of TLR2 and MyD88 was significantly lower in the ZEA and DON groups than in the *L. monocytogenes* group (*p* < 0.05 or *p* < 0.01) (Figure 7A,B). Subsequent measurement of NFκB pathway-related proteins revealed that ZEA and DON treatment also inhibited the expression of TRAF6 and p-TAK1, as well as decreasing phosphorylation levels of IκB and NFκB after *L. monocytogenes* induction (*p* < 0.05 or *p* < 0.01) (Figure 7C). This was consistent with cell measurements.

## 3. Discussion

The co-existence of bacteria and fungi is widespread, and joint exposure can lead to more serious health problems [23,24,25]. ZEA and DON are both common *fusarium* mycotoxins that can cause varying degrees of immunotoxicity effects in animals [26]. It is worth noting that the immunotoxicity induced by these two mycotoxins may be reflected after the organism is infected with pathogenic bacteria. Previously, our team demonstrated that ZEA and DON can inhibit the anti-listeria infection effect mediated by CD4 T cells in mice [27]. In addition, the changes in the innate immune responses of the liver during this process are also worth exploring.

TLR2 is expressed on a variety of immune cells, as well as in non-immune cells, such as hepatocytes [28]. Although not necessarily immune cells, hepatocytes can perform a key innate immune function when pathogenic microorganisms invade the body. Adaptor protein MyD88 in hepatocytes transmits downstream signals in order to stimulate the production of relevant immune products [29]. Studies have shown that deletion of TLR2 and MyD88 increases the susceptibility of the body to pathogenic microorganisms [5,20]. In this study, in vitro treatment with ZEA and DON inhibited TLR2 and MyD88 gene and protein expressions in BRL 3A cells.

Studies have also shown that NOS2 is essential for the clearance of *L. monocytogenes* infection in mice, and NOS2-null mice are more susceptible to *L. monocytogenes* infection than wild-type mice [30,31]. Linda et al. [32] revealed that, in NOS2-deficient mice, granulomas caused by *Bacillus leprae* were 10 times higher than in normal mice. *Escherichia coli* (*E.coli*) enhances nonspecific immune responses by enhancing NOS2 activity in *E. coli*-infected mice [33]. In this study, NOS2 protein expression increased significantly in LTA-treated BRL 3A cells and in mice livers infected by *L. monocytogenes*, which was consistent with previous studies [34]. However, the expression of NOS2 significantly decreased in ZEA and DON treatment groups both in vitro and in vivo. NO synthesized by NOS2 has a direct role in the innate immune defense response against pathogenic microorganisms [34]. Numerous studies have demonstrated that NO can act as a cytotoxic effector molecule when produced by activated macrophages to mediate the inhibition and killing of viruses, bacteria, and other pathogens [35,36]. Boockvar et al. [9] showed that inhibition of NO production in mice infected with *L. monocytogenes* led to an increase in *L. monocytogenes* in the liver and aggravation of the infection. Consistent with previous studies, in this study, in vitro LTA- treated BRL 3A cells and in vivo *L. monocytogenes*-infected mice had significantly increased NO levels, while ZEA and DON treatment significantly inhibited NO production. NOS2 is a key enzyme in the synthesis of NO in innate immunity, so the quantity and quality of NOS2 directly affects the synthesis of NO [37]. In summary, these data indicate that ZEA and DON can reduce the production of NO and increase the bacterial load of liver tissue by inhibiting the expression of NOS2 in LTA-treated BRL 3A cells and in *L. monocytogenes*-infected hepatocytes.

This study demonstrated that ZEA and DON inhibited NO production both in LTA-treated and in *L. monocytogenes*-infected hepatocytes. The resulting reduction in NO levels was matched with an increase in the bacterial loads in liver tissues, thereby aggravating liver damage. TLR2 is a pattern recognition receptor involved in the recognition of lipoproteins from a variety of pathogens, including lipoproteins from Gram-positive bacteria and lipoteichoic acid from Gram-positive bacteria [38,39,40]. TLR2-deficient mice are susceptible to infection with the Gram-positive bacteria *Staphylococcus aureus* [41], while TLR2 activation can promote NO production in hepatocytes, inducing production in a TLR2-MyD88-dependent manner [42]. Measuring the gene and protein expressions of TLR2 and MyD88 in LTA-treated and *L. monocytogenes*-infected hepatocytes in vitro and in vivo after treatment with ZEA and DON revealed a decreased expression of TLR2 and MyD88, which is consistent with previous results. The transcription factor NFκB is involved in many biological processes, including immunity and inflammation. Under normal physiological conditions, NFκB and inhibitors of IκB proteins exist as an inactive complex in the cytoplasm. Once stimulated by TLR ligands, IκB is phosphorylated, targeting the protein for ubiquitination and degradation, causing NFκB to translocate from the cytoplasm to the nucleus and altering the expression of downstream genes, including cytokines and chemokines [40]. TLR2 typically transmits signals through the cytoplasmic adaptor protein MyD88, leading to NFκB activation and proinflammatory gene expression, which protects multicellular organisms from infection [43,44,45]. Gan et al. [46] found that LTA increased the IκBα phosphorylation level and that the activation of NFκB signaling through the recognition of TLR2 upregulated the expression levels of inflammatory genes, cytokines, and chemokines. Upon measuring the expression of key proteins in the NFκB signaling pathway, they found that expression increased significantly in LTA-infected BRL 3A cells in vitro and in the liver tissues of *L. monocytogenes*-infected mice in vivo, which was consistent with some previous studies [8,46]. Interestingly, treatment with ZEA and DON inhibit the expression of key proteins in the NFκB signaling pathway in LTA-treated and in *L. monocytogenes*-infected hepatocytes, while also reducing LTA-induced NFκB nuclear translocation in hepatocytes. This suggests that ZEA and DON can both regulate NO levels in LTA-treated or in *L. monocytogenes*-infected hepatocytes through TLR2/MyD88/NFκB.

## 4. Materials and Methods

### 4.1. Chemicals and Reagent

ZEA, DON and LTA were purchased from Sigma-Aldrich (St. Louis, MO, USA). DMEM medium and fetal bovine serum (FBS) were obtained from Gibcol (Oakland, CA, USA). Cell Counting Kit-8 (CCK8) was obtained from NCM (Suzhou, China). DMSO was purchased from Solarbio (Beijing, China). DAPI was obtained from the Beyotime Institute of Biotechnology (Shanghai, China). The antibodies MyD88, β-actin, GAPDH, TRAF6, p-TAK, IκBα, p-IκBα, NFκB, and p-NFκB were purchased from CST (1:1000, Boston, MA, USA), TLR2 was purchased from Abcam (1:2500, Cambridge, UK), NOS2 was purchased from Proteintech (1:1000, Wuhan, China). All chemicals and reagents were analytical grade. PCR primers were synthesized by Bioengineering. (Shanghai, China)

### 4.2. Cell Cultures

BRL 3A cells were purchased from the American Type Culture Collection (ATCC, Manassas, VA, USA). The BRL 3A cells were cultivated in DMEM and 10% fetal bovine serum, 1% of penicillin, 1 mM glutamine maintained. BRL 3A cells were cultured at 37 °C with 5% CO_2_ and were passaged when adherent cells reached 80–90% confluence. ZEA, DON, and LTA were dissolved in dimethyl sulfoxide (DMSO) and stored at 20 °C.

### 4.3. Cell Proliferation Assay

Cell viability was analyzed using a Cell Counting Kit-8 (CCK8). Cells were plated at a density of 1 × 10^4^ per well in a 96-well plate, after exposure to ZEA at different concentrations (0, 1, 2, 5, 10, 20, and 40 μmol/L), DON at different concentrations (0, 0.1, 0.2, 0.5, 1, 2, and 4 μmol/L) and LTA at different concentrations (0, 0.5, 1, 2, 3, 4, 6, and 8 μgL/mL) for 24 h, CCK-8 solution (10 μmol/L) was added to each well and the culture plate was placed in an incubator for 2–4 h, The absorbance was determined at 450 nm.

### 4.4. Bacterial Cultures

Seventy-two SPF C57BL/6 mice (female, six weeks old, weight 17 ± 1 g) were obtained from the Institute of Comparative Medicine at Yangzhou University (Yangzhou, China). The mouse strain of *L. monocytogenes* EGDe was donated by Professor Weihuan Fang (Zhejiang University, Hangzhou, China) [8].

### 4.5. Animal and Experiment Design

Seventy-two SPF C57BL/6 mice were placed in sterile animal feeding rooms, exposed to 12 h of light per day, fed sterile standard feed and sterile drinking water and allowed to adapt to the environment for at least seven days prior to the experiment. The mice were then randomly divided into four groups of eighteen each: the Control group, the *L. monocytogenes* group, the ZEA + *L. monocytogenes* group, and the DON + *L. monocytogenes* group. Each group of mice was fed in three cages, with six mice in each cage. The ZEA + *L. monocytogenes* group received gavage at 10 mg/kg ZEA/body weight per day, while mice in the DON + *L. monocytogenes* group received gavage at 1 mg/kg DON/body weight per day. The gavage volume of the toxin was set according to the body weight of the mice. Mice in the control group and the *L. monocytogenes* group were given 5% ethyl acetate solution by gavage of the same volume. The toxin was administered orally for 14 days. On day eight, mice were inoculated with bacteria in the tail vein, with each mouse receiving 5 × 10^4^ colony forming units (survival rate 100%) [8].

### 4.6. Liver Pathological Analysis

The mice were euthanized on the third, fifth, and seventh days of bacterial infection, and the mice were dissected to obtain appropriately sized liver tissues, which were fixed, dehydrated, paraffin-embedded, sliced, and stained with Hematoxylin. Eosin staining was used for routine staining. The samples were observed and analyzed.

### 4.7. Bacterial Load Detection

Mice were euthanized on day three, five, and seven post infection. Livers were aseptically harvested and homogenized with sterile PBS, centrifuged at 3000× *g*, and the precipitate was collected. Livers were homogenized in 0.5% Triton-X 100 solution and incubated at room temperature for 30 min. After a gradient dilution in sterile saline, plates were coated at 100 μL per sample, and colonies were counted after growth at 37 °C for 12 h.

### 4.8. NO Level Detection

The treated cells and tissue suspensions were centrifuged at 4 °C for 10 min at 600× *g* after homogenization. The supernatants were then utilized to detect the contents of NO according to the manufacturer’s instruction.

### 4.9. Western Blot Detection of Related Protein Expression

Freshly isolated liver tissue and BRL 3A cells were lysed; the protein concentration was determined using a bicinchoninic acid protein assay kit (Vazyme, Nanjing, China). Equivalent amounts of protein were separated on 10% sodium dodecyl sulfate-polyacrylamide gel electrophoresis and transferred onto polyvinylidene fluoride membranes (Millipore, Danvers, MA, USA). Each membrane was blocked for 2 h using 5% skim milk, incubated overnight with primary antibodies at 4 °C, and then with secondary antibodies for 2 h at room temperature. The primary antibodies were those against: MyD88, GAPDH, TRAF6, p-TAK, IκBα, p-IκBα, NFκB, p-NFκB, TLR2, and NOS2. The membranes were imaged with chemiluminescence. Data quantification analyses were performed using Image J (NIH Image, Stuttgart, Germany).

### 4.10. Quantitative RT-PCR Detection of Related mRNA Expression

Total mRNA extractions were tested for the target genes, such as *TLR2*, *MyD88*, and *GAPDH* by real-time PCR, using the primers listed in Table 1 (Rat primer sequence) and Table 2 (Mice primer sequence).

### 4.11. Immunofluorescence Analysis

The BRL 3A cells were seeded in pre-placed slide plates of a 24-well plate after exposure to different treatment groups for 24 h. After one wash with PBS, cells were fixed with 4% paraformaldehyde at 4 °C for 30 min. After washing again, the cells were treated with 0.5% Triton X-100 at room temperature for 5 min. After washing them again, cells were incubated with 5% BSA sealing solution for 30 min. Next, cells were incubated with NFκB (1:200) primary antibody overnight at 4 °C. After washing once more, the nucleus was stained with DAPI for 15 min. Images were obtained via confocal microscope.

### 4.12. Statistical Analyses

Statistical analyses were performed by GraphPad Prism 5. The data were presented as the mean ± standard deviation (SD). One-way analysis of variance (One-Way ANOVA) was used to determine the significance of differences between groups using SPSS 22.0 software. * *p* < 0.05 and ** *p* < 0.01 were considered statistically significant. Each experiment was performed at least in triplicate.

## 5. Conclusions

**Figure 8:**
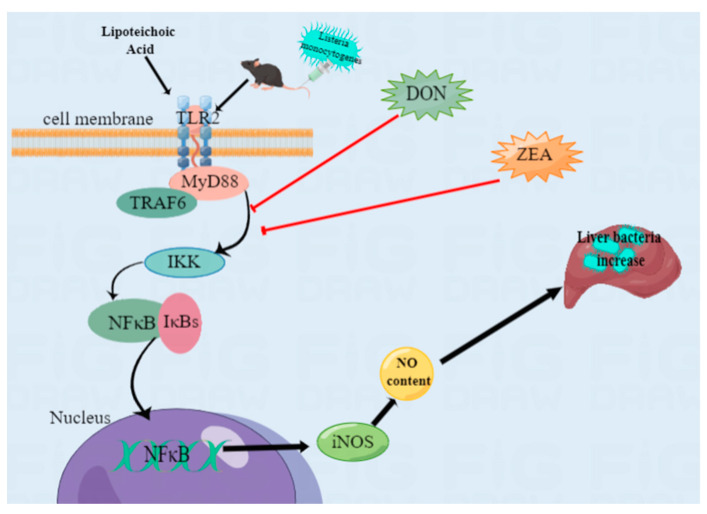
Schematic of mechanisms of innate immune responses to liver after *L. monocytogenes* infection under the influence of ZEA and DON. ZEA and DON can negatively regulate NO through TLR2/MyD88/TRAF6/NFκB, inhibiting the innate immune response of the liver and resulting in increased of liver tissue damage. In the Figure, the red lines denote inhibition and the black line indicates activation. (This image was drawn using Figdraw).

This study indicated that ZEA and DON could inhibit the TLR2/NFκB signaling pathway in LTA-induced BRL 3A cells and in the liver tissue of *L. monocytogenes*-infected mice, thereby inhibiting NFκB activation. This reduced NO levels, and inhibited the innate immune responses of the liver, resulting in an increase in the bacterial load in the liver tissue and aggravating damage to the liver (Figure 8).

## Figures and Tables

**Table 1:** Primer sequence (Rat).

Gene Name	Sequence (5′—3′)
*TLR2*	F: TCTGGAGTCTGCTGTGCCCTTC
	R: GGAGCCACGCCCACATCATTC
*MyD88*	F: ATACGCAACCAGCAGAAACAGGAG
	R: GGTGATGCCTCCCAGTTCCTTTG
*GAPDH*	F: CCTTCATTGACCTCAACTACATG
	R: CTTCTCCATGGTGGTGAAGAC

**Table 2:** Primer sequence (Mice).

Gene Name	Sequence (5′—3′)
*TLR2*	F: CTCCCAGATGCTTCGTTGTTCCC R: GTTGTCGCCTGCTTCCAGAGTC
*MyD88*	F: AGCAGAACCAGGAGTCCGAGAAG
	R: GGGCAGTAGCAGATAAAGGCATCG
*GAPDH*	F: TCAAGAAGGTGGTGAAGCAG
	R: AGTGGGAGTTGCTGTTGAAGT

## Data Availability

The datasets used or analyzed during the current study are available from the corresponding author on reasonable request.
